# Use of oral isotretinoin to treat acne in the public system: a hospital-based retrospective cohort

**DOI:** 10.1590/1516-3180.2018.054405072019

**Published:** 2019-10-31

**Authors:** Letícia Santos Berbert Faria Evaristo, Ediléia Bagatin

**Affiliations:** I MSc. Undergraduate Medical Student, Escola Paulista de Medicina (EPM), Universidade Federal de São Paulo (UNIFESP), São Paulo (SP), Brazil.; II MD, PhD. Dermatologist and Associate Professor, Department of Dermatology, Escola Paulista de Medina (EPM), Universidade Federal de São Paulo (UNIFESP), São Paulo (SP), Brazil.

**Keywords:** Acne vulgaris, Isotretinoin, Dermatology

## Abstract

**BACKGROUND:**

Acne needs to be treated early to prevent negative psychosocial impacts. In severe or moderate forms, which tend to leave scars, oral isotretinoin is the first-line therapy. However, concern about its adverse events, especially in developed countries, delays effective treatment. In contrast, isotretinoin is widely prescribed in Brazilian private clinics.

**OBJECTIVES:**

To describe the use of isotretinoin for treating acne in a Brazilian public hospital, and to analyze whether its prescription is effective or belated.

**DESIGN AND SETTING:**

Retrospective cohort study in a public hospital.

**METHODS:**

Clinical and therapeutic data were obtained from the medical records of patients who were undergoing or had undergone acne treatment with isotretinoin in this hospital’s general dermatology outpatient clinic over the last seven years, up to April 2018.

**RESULTS:**

1526 medical records from patients with acne were analyzed. Isotretinoin was prescribed for 279 patients (18.28%) with mild (1.19%), moderate (57.37%), severe (35.85%) or conglobata (5.57%) forms of acne vulgaris. Sequelae of acne were present at the start of most of these patients’ treatment. An initial daily dose of 20 mg was usually prescribed. The average initial dose/weight ratio was 0.33 mg/kg/day. The average total dose/weight ratio was 127.61 mg/kg. There were only a few cases of laboratory abnormalities.

**CONCLUSION:**

Sequelae of acne at the onset of treatment reveal delayed indication of isotretinoin, which can have negative psychosocial impacts on quality of life. Isotretinoin should be indicated early to prevent this. Its use is supported by its lack of laboratory alterations and controllable adverse events.

## INTRODUCTION

Acne is a chronic and multifactorial disease that involves inflammation of pilosebaceous units.^1^ It mostly affects the face, but may also affect the chest and back, and it presents different cutaneous lesions depending on the severity of the disease. It affects 80% to 90% of adolescents, but can occur at any age, and it has negative psychosocial impacts that may be permanent. For this reason, acne needs to be treated as early as possible, and its management should be directed towards prevention of scars.^2^^,^^3^ Measures such as proper hygiene and sunlight protection form part of the topical and/or systemic therapy for acne, according to the severity of the disease.^4^

For moderate or severe forms of acne that do not respond to conventional therapy, and which tend to leave scars, oral isotretinoin is the first-line therapy. Some authors have stated that isotretinoin should be the first-line drug because of the chronic and unpredictable course of acne.^5^ Isotretinoin is a synthetic analogue of vitamin A that acts epigenetically, through inhibiting sebocyte differentiation and sebaceous gland function and modulating toll-like receptors, regeneration and skin repair. It is used in monotherapy and is highly effective, leading to healing or longstanding remission, prevention and reduction of scars.^6^

Despite the high efficacy of isotretinoin, its use can cause some adverse events, which vary according to the daily dose. The most common and controllable are mucocutaneous conditions (cheilitis, xerophthalmia, nasal dryness and irritative dermatitis); elevated liver enzymes and triglycerides; and changes to cholesterol levels (increased low-density lipoprotein cholesterol and decreased high-density lipoprotein cholesterol). These changes are generally mild and occur early on (after 6 to 8 weeks), and do not have any significant repercussions for the patient’s subsequent follow-up. The effects are transient and reversible, and discontinuation of the treatment is only rarely required.^7^^,^^8^

Other previously reported adverse events that have been matters of controversy, such as depression, suicidal ideation and inflammatory bowel disease, have not been correlated with use of the drug, but with acne itself and not with any treatment.^9^^,^^10^ Teratogenicity is the most serious risk, and extreme caution among women of fertile age is required, including repeated dosages of serum beta-HCG and use of two effective contraceptive methods at least.^11^

Concern regarding adverse events and teratogenicity caused by isotretinoin have mainly been raised in developed countries. These matters of controversy, with associated legal issues, have led to limitations on prescription of isotretinoin, thereby delaying effective treatment and contributing towards a situation in which many people continue to bear acne scars and experience negative repercussions on their quality of life. Such situations may be long-standing and even permanent.^12^^,^^13^^,^^14^

In contrast, isotretinoin is widely prescribed in Brazil for treating acne.^7^^,^^15^ In this country, isotretinoin is freely distributed through the National Health System. According to recent research, isotretinoin was the first choice within the private sector in Brazil for treating moderate and severe acne, for which it was indicated by 76.7% and 94.6% of dermatologists, respectively.^15^ Nonetheless, although isotretinoin is frequently prescribed within the Brazilian private healthcare sector and freely distributed through the public healthcare sector, there is little information on the timing of its prescription and on its effectiveness.

## OBJECTIVE

The aim of this study was to describe the use of isotretinoin for treating acne in a Brazilian public hospital. The possibility of analyzing the efficacy and safety of this drug among a greater number of patients undergoing standardized treatment with adequate follow-up would make it possible to reach a higher level of evidence regarding its use.

## METHODS

This was a hospital-based cohort study conducted in a large referral public center in São Paulo, Brazil. Data were obtained from the electronic system of medical records that was implemented in August 2011 in the general outpatient clinic of the Department of Dermatology of Hospital São Paulo. This is a public hospital within the Brazilian National Health System (Sistema Único de Saúde, SUS) and is the teaching hospital of Escola Paulista de Medicina, Universidade Federal de São Paulo.

The medical records of all patients with a diagnosis of acne up to April 2018 were searched. Individuals who were undergoing or had undergone treatment for acne with isotretinoin were selected for analysis. Information on the patients’ clinical and sociodemographic characteristics was gathered, including gender, age, weight, severity and location of lesions and presence of scars and/or hyperpigmented sequelae; and on the aspects of their therapeutic plans, including the initial and total daily dose, length of treatment, age at the beginning of treatment, dose modification and symptomatic prescription. Data on adverse events like cheilitis, xerosis, xerophthalmia, nasal dryness, increased liver enzymes, alterations in total cholesterol and fractions and increased triglycerides were also analyzed.

The project was approved by the Research Ethics Committee of Universidade Federal de São Paulo/Hospital São Paulo on December 6, 2017 (CEP 1454/2017; CAAE 80370417.7.0000.5505). Even though this was a retrospective study based on medical records, we attempted to contact the participants to ask them to sign a consent statement. Thus, many participants signed this form, but not all of them could be contacted. Nevertheless, given that all the data were used together and were anonymized, we were able to use the data from all participants without any risk of personal identification.

Descriptive statistics were used to analyze the data, and variables were expressed as absolute numbers and percentage values. Laboratory values for the lipid profile and transaminases were compared with reference parameters adapted from Altman et al., 2002.^30^

## RESULTS

In total, 1526 medical records from patients who had been diagnosed with acne were analyzed. Isotretinoin was prescribed for 279 patients (18.28%), of whom 175 (62.72%) were male. Most of them were between 10 and 19 years of age (153; 56.87%) or between 20 and 30 years of age (96; 35.68%). These individuals’ acne began during adolescence in 170 cases (91.39%, out of a total of 186 patients for whom this information was available). Although family histories of acne were often poorly documented (only in 82 medical records), these histories were positive in 25 cases (30.4%).

Isotretinoin was indicated for all levels of severity of acne vulgaris: mild (1.19%), moderate (57.37%), severe (35.85%) and conglobata (5.57%). It was also prescribed for acne in adult women in 12 cases. Data about the lesion site was present in 241 medical records. The face was the site most affected, which showed typical lesions in 235 cases (97.51%), which were concomitant to scars in 148 cases (62.97%) and to post-inflammatory hyperpigmentation in 46 cases (19.57%). The chest and back were affected by acne in 90 patients (37.34%) and 146 patients (60.58%), respectively. In total, sequelae of acne (scars and/or hyperpigmentation) were presented at the start of treatment in 77.1% of the patients, regardless of the site.

An initial daily dose of 20 mg of isotretinoin was prescribed for 215 patients (83.98% of the 256 medical records in which this information was available). The average initial dose/weight ratio was 0.33 mg/kg/day. Among the 279 patients for whom isotretinoin was prescribed at the first medical appointment, 257 patients were followed up subsequently, with further appointments at the outpatient clinic. According to the medical records, most treatments lasted for between 9 and 12 months (57.64%). The average total dose/weight ratio was 127.61 mg/kg.

Mucocutaneous adverse events were the most common type. Most of these patients were given prescriptions for symptomatic treatment, mostly consisting of lip balm and eye drops, together with their prescriptions of isotretinoin. Occurrences of cheilitis were reported in 142 medical records (55.25%), beginning on average after 3.23 months of treatment, with previous prescription of lip balm in 71.12% of these cases. Xerophthalmia was presented by 65 patients (25.29%), starting on average after 4.17 months of therapy, with previous prescription of eye drops in 78.46% of these cases. Presence of xerosis was reported in 40 medical records (15.56%), starting on average after 6.43 months of treatment, with previous prescription of body moisturizer in 10% of these cases. Nasal dryness was the least frequent mucocutaneous event according to the medical records, present in 31 of them (12.06%), beginning on average after 3.93 months of therapy, with previous prescription of nasal saline in 22.58% of these cases.

There were only a few cases of laboratory abnormalities. Regarding the lipid profile, 46 patients (17.89%) reached total cholesterol and/or triglyceride levels above the upper reference limit. Among these, there were some cases with very high values requiring monitoring (9 cases; 3.5%) and one case that would have needed suspension of treatment (0.38%), as specified by Altman et al. (2002).^30^ Regarding hepatic transaminase levels, 29 patients (11.28%) reached aspartate transaminase and/or alanine transaminase levels above the upper reference limit. Among these, there were also a few cases with very high values requiring monitoring (22 cases; 8.56%) and 13 cases that would have needed suspension of treatment (5.05%). Regarding the hemogram, there were no cases of leukopenia or thrombocytopenia, and only one case of mild normocytic normochromic anemia (hemoglobin of 11.8 ­g/­dl), which started after six months of treatment and normalized spontaneously within four months, while this patient was still undergoing treatment.

There were also some reports of other adverse events, which happened rarely or without any relationship established with the drug. After two to three months of treatment, a few medical records reported cases of headache (three cases), myalgia (two), arthralgia (two), “visual blackout” (one), pain and edema in lower limbs (one) and dyspnea (one). After four to five months of treatment, there were a few cases of irritability (three cases) and “sadness” (one case). One case of hypochromia of the lips after six months of treatment was reported, and one case of vaginal dryness after 12 months of treatment. Lastly, there were complaints relating to the gastrointestinal tract in a few medical records: constipation (one case), diarrhea (two cases) and dysphagia (two cases).

Dose reduction due to adverse events was only done in the cases of 24 patients (10.76%). The majority of the medical records showed increases in the doses (102 cases; 45.73%) or maintenance of the doses (68 cases; 30.49%). Some patients had their doses reduced for other reasons (29 cases; 13%), mostly in order to prolong the treatment. Suspension of treatment occurred only in 10 cases, due to clinical symptoms (five cases) or laboratory alterations (five cases).

However, most of the clinical symptoms did not have any clear association with isotretinoin: diarrhea (two cases), headache (one case), “visual blackout” (one case, in which there was also a tendency towards elevated lipid profile and a family history of dyslipidemia) and myalgia, low back pain and lower-limb pain (one case). Regarding laboratory alterations, these were four cases with transaminase levels that would imply drug withdrawal, as specified by Altman et al. (2002),^30^ i.e. aspartate transaminase > 80 U/l and/or alanine transaminase > 62 U/l. One of these cases just needed close monitoring, and isotretinoin was reintroduced after two weeks of suspension.

## DISCUSSION

In comparison with a recent analysis on isotretinoin prescription in Brazilian private clinics, which showed that 76.7% and 94.6% of dermatologists would prescribe the drug in cases of moderate and severe acne respectively,^15^ indication of isotretinoin in the public hospital studied here over the period analyzed was only implemented for a small percentage (18.28%) of the patients diagnosed with acne.

This was an unexpected result, since the drug is provided free-of-charge through the Brazilian National Health System and because people who seek care in public hospitals generally present the disease with longer evolution and greater severity, such that isotretinoin would be the first-choice treatment. However, public hospitals are also the first places for treatment sought by people experiencing financial difficulties and social problems, and in those cases, acne may present a mild level of severity according to the perceptions of both the patient and the family. These patients receive topical treatments with or without association with oral antibiotics, which may be enough to properly control the disease.

It should be noted that prescription of topical and oral antibiotics for treating acne has been reviewed. It was concluded that such prescriptions should follow recommendations for limited and rational use, given the increasing degrees of bacterial resistance, which are leading to repercussions regarding upper respiratory infections.^16^^,^^17^ Furthermore, the existence of wrong and even frightening information about isotretinoin, especially among people with lower education, may interfere with patients’ and their families’ acceptance of this medication. This would corroborate the low percentage of prescription that was seen in the present study.^18^

Among the patients of the present study, isotretinoin was more prescribed for males, and for adolescents and young adults, and this finding agreed with data in the literature.^15^^,^^19^ In the vast majority of cases, acne started in adolescence and consisted of acne vulgaris. Unfortunately, only a few of the medical records gave any information about presence or absence of a family history of acne. Investigation of this matter needs to be emphasized in obtaining the anamnesis, because this information is important and can provide evidence of the severity and evolution of acne.^19^

The results regarding types of acne corroborated the data in the literature, since more than 90% of the patients treated with ISO had moderate or severe acne. It was observed that almost all the patients presented acne on the face, and some also on the chest and the back.

Scars were commonly present, and were already visible at the beginning of the treatment, particularly on the face. This revealed that there had been delays in drug indication.^14^ Such delays lead to psychosocial repercussions that can be long-lasting and may have a negative impact on these individuals’ quality of life. For this reason, emphasis is placed on the importance of early prescription.^4^

Most of the patients undergoing treatment at the outpatient clinic were properly followed up, in accordance with guidelines that have been published since the 1990s.^19^^,^^20^ This is important, because isotretinoin is a teratogenic drug with possible side effects.^11^

On average, the therapeutic regimens used an initial daily dose of 0.33 mg/kg. The dose most commonly used was 20 mg, which was lower than what is recommended in the package insert, which is 0.5-1.0 mg/kg/day. This dosage level was probably adopted because of the impossibility of monthly follow-up in this public hospital, given the high level of demand for appointments from patients. It is known that a low starting dose prevents the initial exacerbation that occurs in some cases, which scares patients and may cause poor adherence to the treatment.^21^ In addition to greater patient adherence, it has been shown in several studies that a low daily dose regimen has the same efficacy, but with fewer adverse events that are dose proportional, and higher satisfaction among the patients.^22^^,^^23^^,^^24^

Isotretinoin was well tolerated by many patients, as shown by the gradual increase in the daily dose that they tolerated. For other patients, the dose of 20 mg/day was maintained until the end of treatment, thus extending the duration of the treatment to an average of 9 to 12 months, instead of the 4-6 months, as has been recommended since isotretinoin was introduced in the market, more than 30 years ago.^25^ Nevertheless, the total dose was 127.61 mg/kg, which was within the range recommended in the package insert, i.e. 120-150 mg/kg, and also in line with the opinions of some authors like Rademaker^26^ and Tan et al.^20^

All the patients who completed the treatment were referred for maintenance treatment, as recommended in the literature. Maintenance treatment using several topical products, except antibiotics, has been recommended for periods of 6 to 12 months after the disease has been resolved.^27^^,^^28^

Mucocutaneous adverse events of mild intensity occurred, as expected, and were controllable through symptomatic treatment. This was prescribed at the same time as isotretinoin, for prevention of the most common mucocutaneous adverse events: cheilitis, xerophthalmia, nasal dryness and xerosis. This management approach was fully in accordance with the well-known recommendations.^29^ It needs to be borne in mind that appointments were made every three months and, for this reason, the medical records did not precisely define the time at which the symptoms began, which tended to be earlier than what was seen.

There were very few cases of laboratory abnormalities caused by use of isotretinoin, and even fewer cases that required dose reduction or suspension of treatment due to adverse events. The criteria for such decisions were the reference values defined by Altman et al. (2002).^30^ These findings are consistent with the data in the recent literature, in which reduced levels of monitoring are recommended, i.e. only for tests that show significant changes, thereby diminishing the associated costs.^8^^,^^31^^,^^32^^,^^33^

There were no reported cases of depression, suicide or inflammatory bowel disease. This confirmed the findings from population-based studies, which have not shown any association of these diseases with use of isotretinoin.^9^^,^^10^ It is known that depressive states are related to acne itself,^34^ while inflammatory bowel disease is associated with many chronic inflammatory diseases and with use of antibiotics, including those used in treating acne.^35^

The present study had potential limitations. Firstly, it was based on medical records, which are not always filled out in detail. Secondly, it analyzed patients from a single public hospital, which may not have reflected the reality of the public system as a whole. Lastly, since it was a cross-sectional study, no “cause and effect” relationship could be established.

## CONCLUSION

The large presence of acne sequelae at the onset of treatment revealed the existence of delayed indication of drug treatment, which may lead to scars and may have a strong negative psychosocial impact on quality of life. The wide indication of isotretinoin for treating moderate forms of acne was an important measure for avoiding this scenario. The results from this study are in agreement with the data from the most recent studies in the literature: low daily dose, total dose according to package insert recommendations, common side effects and laboratory alterations that were few in number, mild and controllable. Further studies like this one are necessary in order to analyze whether recent data on oral isotretinoin therapy for acne has indeed been applied to clinical practice.
